# A Bayesian approach for estimating the uncertainty on the contribution of nitrogen fixation and calculation of nutrient balances in grain legumes

**DOI:** 10.1186/s13007-024-01261-9

**Published:** 2024-09-02

**Authors:** Francisco Palmero, Trevor J. Hefley, Josefina Lacasa, Luiz Felipe Almeida, Ricardo J. Haro, Fernando O. Garcia, Fernando Salvagiotti, Ignacio A. Ciampitti

**Affiliations:** 1 Department of Agronomy, Kansas State University, 1712 Claflin Rd, Manhattan, KS 66506 USA; 2 Department of Statistics, Kansas State University, 205 Dickens Hall, 1116 Mid-Campus Drive North, Manhattan, KS 66506 USA; 3 EEA Manfredi INTA, Ruta 9 km 636, Manfredi, Córdoba 5988 Argentina; 4 Former IPNI Latin America Southern Cone Program, Private Consultant, Balcarce, Buenos Aires 7620 Argentina; 5 Crops, Soils and Water Management Group, EEA Oliveros INTA, Ruta 11 km 353, Oliveros, Santa Fe 2206 Argentina; 6 Consejo Nacional de Investigaciones Científicas y Técnicas (CONICET), Buenos Aires, 1033 Argentina

**Keywords:** Delta method, Bootstrapping, N balance, Roots

## Abstract

**Background:**

The proportion of nitrogen (N) derived from the atmosphere (Ndfa) is a fundamental component of the plant N demand in legume species. To estimate the N benefit of grain legumes for the subsequent crop in the rotation, a simplified N balance is frequently used. This balance is calculated as the difference between fixed N and removed N by grains. The Ndfa needed to achieve a neutral N balance (hereafter $\theta$) is usually estimated through a simple linear regression model between Ndfa and N balance. This quantity is routinely estimated without accounting for the uncertainty in the estimate, which is needed to perform formal statistical inference about $\theta$. In this article, we utilized a global database to describe the development of a novel Bayesian framework to quantify the uncertainty of $\theta$. This study aimed to (i) develop a Bayesian framework to quantify the uncertainty of $\theta$, and (ii) contrast the use of this Bayesian framework with the widely used delta and bootstrapping methods under different data availability scenarios.

**Results:**

The delta method, bootstrapping, and Bayesian inference provided nearly equivalent numerical values when the range of values for Ndfa was thoroughly explored during data collection (e.g., 6–91%), and the number of observations was relatively high (e.g., $\ge 100$). When the Ndfa tested was narrow and/or sample size was small, the delta method and bootstrapping provided confidence intervals containing biologically non-meaningful values (i.e. < 0% or > 100%). However, under a narrow Ndfa range and small sample size, the developed Bayesian inference framework obtained biologically meaningful values in the uncertainty estimation.

**Conclusion:**

In this study, we showed that the developed Bayesian framework was preferable under limited data conditions ─by using informative priors─ and when uncertainty estimation had to be constrained (regularized) to obtain meaningful inference. The presented Bayesian framework lays the foundation not only to conduct formal comparisons or hypothesis testing involving $\theta$, but also to learn about its expected value, variance, and higher moments such as skewness and kurtosis under different agroecological and crop management conditions. This framework can also be transferred to estimate balances for other nutrients and/or field crops to gain knowledge on global crop nutrient balances.

**Supplementary Information:**

The online version contains supplementary material available at 10.1186/s13007-024-01261-9.

## Background

The biological nitrogen (N) fixation is an essential process in legume species. This process is highly relevant in agroecosystems because it represents a sustainable strategy to possibly increase soil N stock [1], reducing the dependence on N fertilizers and thus minimizing agriculture's environmental footprint [2]. The fixed N is stored in crop tissues until harvest, where a fraction of this N is exported with grains and other remains in the field as stover. The proportion of N that comes from the N fixation process, with respect to the crop N demand, is termed as N derived from the atmosphere (Ndfa). This quantity is typically computed as:

$${\text{Ndfa}} = { }\left[ {\frac{{Fixed{ }~N{ }~\left( {kg{ }~ha^{ - 1} } \right)}}{{N{ }uptake{ }~\left( {kg{ }~ha^{ - 1} } \right)}}} \right] \cdot 100{\text{\% }}$$

There is a general consensus in grain legume studies to compare the Ndfa with the proportion of N allocated to the grains (i.e., the N harvest index, NHI) for estimating N gains or losses in the cropping system [3, 4]. When the Ndfa (relative N input) is greater than the NHI (relative N output), legumes are expected to contribute with N to the overall soil N balance. The N balance of legume crops is usually estimated as the difference between the quantity of fixed N by the crop and removed N by harvestable organs (both expressed in kg ha^−1^). This is a simplification to calculate the N balance in agroecosystems because other N inputs (synthetic N fertilizers, manures, atmospheric N depositions, irrigation water) and outputs (leaching, volatilization, denitrification, surface runoff) are not considered [5, 6]. However, for the purposes of this study, it is defined that: (i) when the contribution of the belowground N (N in roots and rhizodeposition) is excluded, the N balance is referred to as partial N balance (PNB); and (ii) when this plant fraction is included, it is termed total N balance (TNB).

**Fig. 1:**
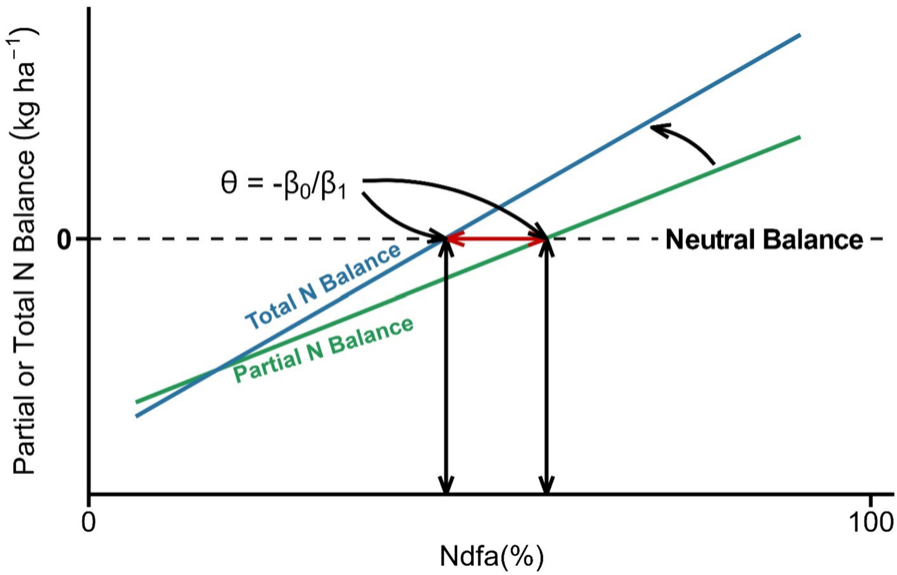
Illustration of the estimation of the Ndfa (proportion of the total crop N derived from the atmosphere) needed to achieve a neutral partial or total N balance ($\theta$). That quantity called $\theta$ is calculated as a function of the intercept (${\beta }_{0}$) and of the slope (${\beta }_{1}$) of the common linear regression model for partial or total N balance ($y$) as a function of Ndfa ($x$)

The Ndfa needed to achieve a neutral PNB or TNB can be estimated as a function of Ndfa [7, 8]. Usually, the relationship between Ndfa and PNB or TNB is described with the simple linear regression model (Fig. 1):

1 $${y}_{i} = {\beta }_{0} + {\beta }_{1}{x}_{i} + {\varepsilon }_{i},$$

where ${y}_{i}$ is the PNB or TNB (in kg ha^−1^) for the *i*th observation, ${x}_{i}$ is the *i*th observation of Ndfa (predictor variable), ${\beta }_{0}$ (intercept) is the expected PNB or TNB when the crop did not fix N (i.e., when ${x}_{i}=0$), ${\beta }_{1}$ (slope) is the change in PNB or TNB per unit of Ndfa (note that our previous knowledge on the subject allows us to assume ${\beta }_{1}\ge 0$), and ${\varepsilon }_{i}$ is the residual error. After fitting the model, the expected value of PNB or TNB is set to zero, that is $E\left({y}_{i}\right)=0$. By doing so, it is said that PNB or TNB are expected to be neutral. Then, according to Eq. 1, the Ndfa to get a neutral PNB or TNB can be determined by finding the $x$ value when ${E(y}_{i})=0$. We label this quantity $\theta$, and it can be calculated according to

2 $$\theta = {\raise0.7ex\hbox{${ - \beta_{0} }$} \!\mathord{\left/ {\vphantom {{ - \beta_{0} } {\beta_{1} }}}\right.\kern-0pt} \!\lower0.7ex\hbox{${\beta_{1} }$}},$$

as it is shown in Fig. 1.

For modeling purposes, it is important to consider that the Ndfa is a proportion that can only take values between 0 and 100. Likewise, $\theta$ is the Ndfa to achieve a neutral N balance (PNB or TNB) that also can only take values between 0 and 100. Thus, $0\le \theta \le 100$. After $\theta$ is estimated using Eq. 2, its uncertainty must be quantified to enable formal statistical inference. Perhaps the two most common approaches to quantify the uncertainty are the delta method [9] and bootstrapping [10], which enable the calculation of standard errors and confidence intervals on the estimates for $\theta$. The delta method is an asymptotically (large sample size) based technique that implements Taylor series approximation to approximate the variance of a function of a random variable. On the other hand, bootstrapping is a computational technique based on a resampling of the observed data. Alternatively, a third approach, Bayesian inference, may also be used to estimate $\theta$ and quantify its uncertainty.

A Bayesian framework is convenient for scenarios with a limited number of observations, especially when previous information exists [11]. Bayesian inference is a statistical technique based on Bayes and Laplace’s work early in the 1700’s, however, in the past 40 years rapid computational advancements have made Bayesian methods usable and accessible to scientists [12]. Bayesian inference appears to be underutilized in legume research for estimating $\theta$ and quantifying its uncertainty [7, 8, 13, 14]. In this article we will present how Bayesian inference, the delta method, and bootstrapping effectively address the uncertainty quantification of $\theta$ when the data at hands contain valuable information. Furthermore, we will depict how Bayesian inference can be effective to address such problems under limited data conditions.

In most cases, $\theta$ is estimated without quantifying or considering the uncertainty surrounding the estimate [7, 8, 13, 14]. This point estimate approach, while useful, does not allow for formal statistical inference which is needed to obtain reliable scientific conclusions. Furthermore, it is important to select an appropriate statistical technique that matches the biological underlying assumptions of the range of values that the variable can take. In this article we demonstrate the delta method and bootstrapping, and we describe the development of novel Bayesian framework to quantify the uncertainty of the Ndfa needed to attain neutral N balance in legume crops ($\theta$). We hypothesized that under limited data conditions, the developed Bayesian framework provided better uncertainty estimations of $\theta$ than the delta method and bootstrapping, while minor differences were expected among the three methods under greater data availability.

The aims of this study were to (i) develop a Bayesian framework to quantify the uncertainty on the Ndfa needed to achieve neutral PNB or TNB in grain legume species, and (ii) contrast the use of this framework with the delta method and bootstrapping under different scenarios of data availability. The developed Bayesian framework can expand a new study niche in agriculture, offering opportunities for parameter estimations, formal statistical inference, uncertainty quantification and propagation, among other applications. Furthermore, this article also serves as a practical guide for Bayesian non-practitioners to apply Bayesian inference in other areas of study within the field of agriculture. We present a case study with the sole objective of illustrating a potential use of this statistical framework.

## Materials and methods

**Fig. 2:**
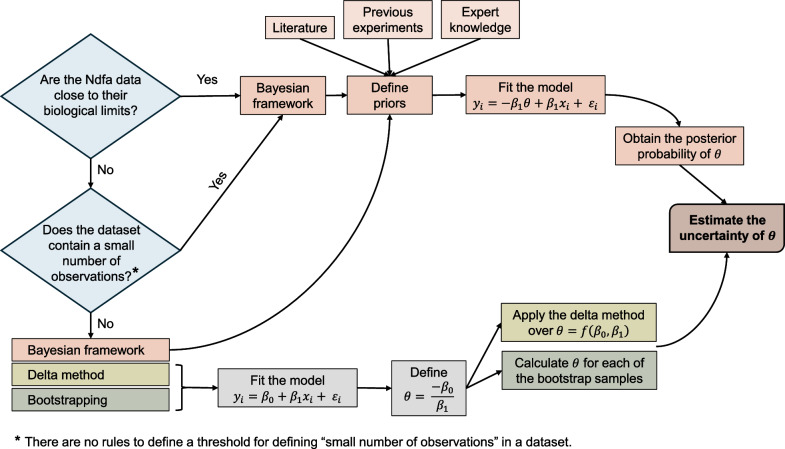
Flowchart depicting the workflow implementing in this study and showing the cases where it is worth applying the developed Bayesian framework

We illustrate the three methods (delta method, bootstrapping, Bayesian inference) for quantifying the uncertainty on $\theta$ by retrieving the data from [13]. The workflow implemented in this study is depicted in the flowchart presented in Fig. 2. Furthermore, Fig. 2 provides information to future users to decide in which cases apply the Bayesian framework developed in this article. Since there is no rule for defining when datasets are small, overall, it would be justified running the proposed Bayesian framework in cases where information is available to define priors and/or the delta method and/or bootstrapping provide unreliable uncertainty estimations (e.g. $\theta >100\%~or~\theta <0\%$).

### Data collection and description

The variables (Ndfa, fixed N, seed N) were collected in chickpea (*Cicer arietinum* L.), common bean (*Phaseolus vulgaris* L.), cowpea (*Vigna unguiculata* L.), faba bean (*Vicia faba* L.), field pea (*Pisum sativum* L.), lentil (*Lens culinaris* Medik), white lupin (*Lupinus albus* L.), blue lupin (*Lupinus angustifolius* L.), and peanut (*Arachis hypogaea* L.). A similar literature search was conducted to retrieve the proportion of N that is allocated to the roots and rhizodeposition with respect to the total N in the plant (above + belowground N). For more details about the criteria to select papers for the database see [13]. We also included unpublished data for peanut in the current study.

### Variable descriptions and calculations

The authors in [13] studied belowground N contributions retrieving the information from articles implementing physical recovery and ^15^N-labelling techniques for quantifying belowground N [15]. With the collected information, Palmero et al. [13] calculated a root factor as:

3 $$Root\, Factor = 1 + \frac{Below\, ground\, N}{Above\, ground\, N},$$

where *Below ground N* and *Above ground N* refer to the proportions of N found below and above the ground, respectively, relative to the total plant N demand. For instance, assume a scenario where the total N in a crop (considering above and belowground structures) is 125 kg ha^−1^, but this value is unknown. What is known is the amount of N (kg ha^−1^) in the aboveground structures of the crop and the proportion of N allocated to the roots relative to the total N in the crop. Assuming that the aboveground N is quantified at 100 kg ha^−1^ and the root N allocation is 20%. Therefore, we know that 100 kg of N ha^−1^ represents 80% of the total N in the crop. Therefore, by calculating the root factor, and applying it to estimate the total N uptake (above + belowground structures), we have the following:

$$Root\,Factor{ } = { }1{ } + { }\frac{0.20}{{0.80}} = 1.25$$

$$Total\,N\,in\,the\,crop\,\left( {kg{ }~ha^{ - 1} } \right) = { }100{ }~kg{ }~ha^{ - 1} \cdot { }1.25 = 125~{ }kg{ }~ha^{ - 1}$$

Thus, the root factor allows the incorporation of the N allocated to the roots when calculating the total fixed N by a crop (see Eq. 5).

The Ndfa (%) values, representing the proportion of total aboveground N derived from the N fixation process, were obtained through a literature review only for aboveground parts of the crop. The fixed N (kg ha^−1^) can be calculated by excluding the contribution of N from roots and rhizodeposition as follows:

4 $$Fixed\,Aboveground\,N\,\left(kg~{ha}^{-1}\right)= Total\,Aboveground\,Uptake\,N\,\left(kg~{ha}^{-1}\right)\cdot \frac{Ndfa (\%)}{100}.$$

In addition, assuming Ndfa did not differ between above- and below-ground structures [16], the Ndfa estimated for the aboveground tissues can be used to estimate the fixed N considering the belowground N contribution according to Eq. 3 and Eq. 4 as

5 $$Total\,Fixed\,N\,\left(kg~{ha}^{-1}\right)= Total\,Aboveground\,Uptake\,N\,\left(kg~{ha}^{-1}\right)\cdot Root\,Factor\cdot \frac{Ndfa (\%)}{100}.$$

Lastly, the Fixed Aboveground N and Total Fixed N can be used to calculate the PNB and TNB, respectively, as follow:

6 $$PNB~(kg~{ha}^{-1}) = Fixed\,Aboveground\,N\,(kg~{ha}^{-1}) - Seed\,N\,(kg~{ha}^{-1}),$$

7 $$TNB\,(kg {ha}^{-1}) = Total\, Fixed\,N\,(kg~{ha}^{-1}) - Seed\,N\,(kg~{ha}^{-1}).$$

If either of these balances are positive, it means that the fixed N (excluding (Eq. 6) or including (Eq. 7) belowground N contribution) is greater than the N exported in seeds and a net soil N input occurs, resulting in a positive N balance. On the other hand, negative values indicate that the fixed N was not enough to compensate the N exported in seeds and a net soil N reduction takes place, resulting in a negative N balance.

### Statistical models

In this section, we will provide details about the different approaches to estimate the parameter of interest and introduce a few modifications in the original model [7, 8] to incorporate previous knowledge in the statistical model.

#### Regression model

First, we used a simple linear regression model for PNB or TNB as a function of Ndfa. We introduced this model in Eq. 1 as

$${y}_{i} = {\beta }_{0} + {\beta }_{1}{x}_{i} + {\varepsilon }_{i},$$

where ${y}_{i}$, ${x}_{i}$, ${\beta }_{0}$, ${\beta }_{1}$, and ${\varepsilon }_{i}$ have the same interpretation than that mentioned in the background section for Eq. 1. This model has a deterministic part, ${\beta }_{0} + {\beta }_{1}{x}_{i}$, and a random part, ${\varepsilon }_{i}$, which is usually assumed that ${\varepsilon }_{i}\sim N(0, {\sigma }^{2})$. The Ndfa needed to achieve neutral N balances in grain legume species (called $\theta$) can be calculated as a function of ${\beta }_{0}$ and ${\beta }_{1}$. The delta method and bootstrapping can be implemented to quantify the uncertainty of $\theta$.

The delta method allows us to approximate sampling distributions for functions of random variables. Since both ${\beta }_{0}$ and ${\beta }_{1}$ have their own variance estimation and $\theta$ is a function of the $\beta {\prime}s$, the delta method can be applied to estimate the variance of $\theta$ [17]. Then, under the assumption that the sampling distribution of the ratio $\frac{-{\beta }_{0}}{{\beta }_{1}}$ is asymptotically normally distributed, the approximated variance of $\theta$ (obtained via delta method) can be used to construct confidence intervals.

The bootstrapping technique utilizes the *plug-in* principle to estimate the population distribution based on the empirical distribution of the observed data [10]. Applying this computational technique to a linear model consists of taking *K* random samples (with replacement) of the same size as the original data (*n*), and then fitting the linear model to each of the *K* samples of size *n*. Finally, the K estimates are utilized to construct the confidence interval of $\theta$. In this study, *K* was equal to 10,000.

#### Bayesian inference

Bayesian inference offers an alternative to solve the challenges found when implementing the delta method and bootstrapping for this particular study. Through Bayesian inference, the estimation of the model parameters and their variability can be regularized by including previous knowledge into the model [18]. For example, in Fig. 1, values of $\theta$ greater than 100 are not possible. Therefore, it is realistic to incorporate this assumption into our linear regression model. Up to this point, the model as presented in Eq. 1 can be implemented under any framework. However, a Bayesian framework is needed in some situations because it allows us to incorporate information about the model parameters in the form of probability distributions, known as prior distributions (regulator; Table 1).

**Table 1:** Glossary of terms and definitions

Term	Definition
Moments	A set of values used to quantify characteristics of a probability distribution, such as, its mean (expected value) and variance. Moments describe the shape, location, and spread of a probability distribution
Expected value	The mean of a random variable weighted according to the probability distribution. It is the first moment of the probability distribution of a random variable. It is represented as E(.)
Variance	The second central moment of the probability distribution of a random variable. It measures the degree of spread of a distribution around its mean
Prior distribution	An assumed function that maps the probability for a specific model parameter, that is independent of the data to be analyzed (e.g., $\theta \sim N(\text{0,2})$). The natural regulator in Bayesian models
Hyperparameter	The parameter that defines the prior distribution that is assumed fixed and known (e.g., 0 and 2 in a prior distribution for $\theta$ that is $\theta \sim N(\text{0,2})$)
Likelihood	A function that maps the probability or density of the model parameters given the observed data. It is the link between the data and the posterior distribution of the parameters in Bayesian models
Posterior distribution	Probability distribution of the parameters after observing (given) the data
Marginal posterior	Probability distribution of a single random variable in a Bayesian model that is conditional only on the data
Regularization	The process of constraining a statistical inference problem (i.e., penalization or shrinkage)
Regulator	Prior, penalty, or constraint

As described, the regression model in Eq. 1 does not allow to incorporate knowledge about the $\theta$, because $\theta$ is not directly but indirectly present in Eq. 1 by utilizing ${\beta }_{0}$ and ${\beta }_{1}$ (Eq. 2; Fig. 1). In Bayesian inference any unobserved quantity is considered a random variable. Therefore, the model parameters ${\beta }_{0}$, ${\beta }_{1}$ are random variables under the Bayesian paradigm. Furthermore, a function of a random variable is also a random variable. We showed that $\theta = \frac{{-\beta }_{0}}{{\beta }_{1}}$. Therefore, $\theta$ becomes a parameter that models the proportion of N that the legume crop has to fix (Ndfa) to achieve a neutral N balance. Using the expression $\theta = \frac{{-\beta }_{0}}{{\beta }_{1}}$, we can write ${\beta }_{0}$ as a function of $\theta$ and ${\beta }_{1}$ represented as ${\beta }_{0} = {-\beta }_{1}\theta .$ Now, we can use the last equality to plug it in Eq. 1. Thus, the original linear model can be re-written as

8 $${y}_{i}= {-\beta }_{1}\theta + {\beta }_{1}{x}_{i} + {\varepsilon }_{i}$$

where ${y}_{i}$ is the PNB or TNB (kg ha^−1^) for the *i*th observation, ${x}_{i}$ is the *i*th observation of Ndfa, ${\beta }_{1}$ represents the change in PNB or TNB per unit of Ndfa, $\theta$ is the Ndfa required to achieve a neutral N balance, and ${\varepsilon }_{i}$ is the residual error. Since the framework we propose in this study is Bayesian, we need to provide priors (also called parameter models) for the model parameters in Eq. 8. We have assumed that ${\varepsilon }_{i}\sim N\left(0, {\sigma }^{2}\right).$ Therefore, ${\sigma }^{2}$ ─or the standard deviation ($\sqrt{{\sigma }^{2}}=\sigma$)─ is a parameter to be estimated as is ${\beta }_{1}$ and $\theta$. The parameter models (priors) selected on this study were:

9 $${\beta }_{1} \sim gamma(1.6, 0.8),$$

10 $$\theta \sim beta(\alpha , \beta ),$$

11 $$\sigma \sim gamma\left(2.5, 0.05\right).$$

The numerical values within the parentheses in Eq. 9–11 are the parameters that shape the probability distribution. In Bayesian inference, these parameters (e.g., 1.6 and 0.8 in Eq. 9) are referred to as hyperparameters (Table 1). In this study, we uniquely determined the values for the hyperparameters for the beta distribution ($\alpha$ and $\beta$) for each species, and their values are presented in Table 2. Setting the hyperparameters allows us to define the expected value and variance for the prior probability distributions. The computed expected values were $E\left({\beta }_{1}\right)=\frac{1.6}{0.8}$, $E\left(\sigma \right)=\frac{2.5}{0.05}$, and $E\left(\theta \right)= \frac{\alpha }{\alpha +\beta }$. More details related to parameters and probability distributions selected as prior are provided in the subsequent sections.

**Table 2:** Moments and hyperparameters for the prior probability distribution of $\theta$ parameter for Partial N Balance (PNB) and Total N Balance (TNB)

Species	Moments				Probability distribution	Hyper parameters	
	PNB		TNB				
	E($\theta$)	Var($\theta$)	E($\theta$)	Var($\theta$)		PNB	TNB
Blue lupin	0.76	0.0066	0.59	0.0098	Beta	(20.26, 6.33)	(13.94, 9.51)
Chickpea	0.75	0.0078	0.42	0.0117	Beta	(17.16, 5.76)	(8.24, 11.41)
Common bean	0.69	0.0368	0.53	0.0553	Beta	(3.30, 1.45)	(1.85, 1.65)
Cowpea	0.61	0.0064	0.28	0.0096	Beta	(22.06, 14.11)	(5.82, 14.48)
Faba bean	0.84	0.0826	0.56	0.0124	Beta	(12.99, 2.53)	(10.58, 8.28)
Field pea	0.64	0.0152	0.44	0.0228	Beta	(9.04, 5.00)	(4.34, 5.45)
Lentil	0.72	0.0239	0.44	0.0356	Beta	(5.35, 2.10)	(2.56, 3.29)
Peanut	0.65	0.0162	0.57	0.0243	Beta	(8.47, 4.62)	(5.21, 3.84)
White lupin	0.82	0.0019	0.57	0.0029	Beta	(62.52, 14.06)	(47.94, 35.35)

The moments were calculated using the collected information about NHI and the hyperparameters were obtained by moment matching based on the previously calculated moments

#### Informative priors

The Bayesian framework consists of three core steps: (i) determining the likelihood function; (ii) capturing the knowledge about the parameters in the statistical model through the prior distribution; and (iii) combining the likelihood and the prior applying the Bayes’ theorem to obtain the posterior distribution of the model parameters (Table 1; Supplementary Note 1). The priors influence the posterior distribution of the model parameters, as the posterior is a balance between the data, the likelihood, and the priors [19, 20]. However, the impact of the priors on the posterior is usually reduced as sample size increases. Bayesian statistics allow us to incorporate previous scientific knowledge into our model through the use of priors. The information (data or expert knowledge) used for specifying the hyperparameters must be independent from the data used to fit the parameters of the model [18]. In this study, we employed informative priors for the model parameters based on independent information collected from previous studies.

Because a greater Ndfa means a larger proportion of the total crop N being fixed, higher Ndfa results in larger N balances, making ${\beta }_{1}$ to take positive values [7, 8, 14]. The standard deviation parameter ($\sigma$) is the positive square root of the variance, and thus, can take only positive real numbers. The gamma distribution is a continuous distribution of the positive real numbers that can take different shapes. Therefore, we chose the gamma distribution to represent our prior knowledge of ${\beta }_{1}$ and $\sigma$. The parameter $\theta$ represents a proportion, indicating the Ndfa required to achieve a neutral N balance. Therefore, $\theta$ can take only positive values between 0 and 1 (or 0 and 100 if scaled). Since the beta distribution models continuous random variables that can take values between 0 and 1, we selected it as prior for $\theta$. Furthermore, the beta distribution has flexible shapes, which is not the case of a standard uniform distribution. Selecting beta as prior distribution for $\theta$ restricts its values, which are in fact delimited by the nature of the Ndfa concept to be $0\le \theta \le 1$. This exemplifies how priors can behave as regulators in Bayesian models [18].

The hyperparameters for the beta distribution used in Eq. 10 for $\theta$ were defined based on previous literature to best represent our prior knowledge of that parameter. However, no information was directly available for the $\theta$. Thus, bringing Eq. 4 and Eq. 6 and considering that $\theta$ represents the Ndfa value when PNB (or TNB) is equal to zero:

$$PNB \left( {kg ha^{ - 1} } \right) = Total\, Aboveground\, Uptake\, N\, \left( {kg ha^{ - 1} } \right) \cdot \frac{Ndfa \left( \% \right)}{{100}} - Seed\,N \,\left( {kg ha^{ - 1} } \right),$$

$$\frac{Ndfa \left( \% \right)}{{100}} = NHI$$

This result indicates that the PNB equals zero when the Ndfa is equal to the N harvest index or NHI [3, 4]. This calculation uses PNB rather than TNB because most current scientific literature calculates NHI without considering below-ground biomass contribution.

We conducted a literature review to collect information about the NHI of the nine legumes species included in this study. This review was independent from that utilized by [13]. The literature search was conducted through Google Scholar^®^, Scopus^®^, and Web of Science^®^ search engines (last search on August 8, 2023) using the following keywords: ("legume scientific name" OR "legume vulgar names") AND ("nitrogen harvest index" OR "NHI" OR "N harvest index"). We retrieved a total of 153 articles (excluding duplicates). The selection criteria were: (i) the experiments were performed in field conditions; (ii) NHI has been reported, and calculated excluding roots; and (iii) management information (e.g., N rate, sowing date, irrigation, insect, and pest management) and potential stress factors (e.g., drought, heat, nutrient deficiency) were reported. If the crop performance was severely affected by management practices or stress, the study was not included to avoid NHI values < 0 or > 1. In addition, the study must not have been included in the previous review process used to build the original database to ensure data independence to build the priors for ${\theta }_{PNB}$ and ${\theta }_{TNB}$. Ultimately, out of the pooled of articles, a total of 42 studies were included in the analysis (https://figshare.com/s/60a9cf527ecb9de02166).

We used the NHI values collected for each species to calculate its mean and variance, which represent the mean and the variance of the Ndfa required for a neutral PNB, herein termed as ${\theta }_{PNB}$. Then, the mean and the variance were used to calculate the parameters ($\alpha$ and β) of the beta distribution via moment matching (Supplementary Note 2), which was used as prior distribution for each of the studied species (Table 2). For the Ndfa to achieve a neutral TNB, we reduced the expected value of the Ndfa in a proportion equivalent to the proportion of N that is allocated to root according to previous information [13, 21]. Given the low certainty of this prior information, we increased the variance of the prior distribution by 50% to account for this uncertainty. Finally, we used the recalculated mean and variance to calculate the hyperparameters of the beta distribution for $\theta$, now referred as ${\theta }_{TNB}$ (Table 2). Supplementary Fig. 1 illustrates the discrepancies between prior probability distributions of ${\theta }_{PNB}$ and ${\theta }_{TNB}$.

### Model fitting and parameters of the posterior probability distributions

The model presented in Eq. 8 to Eq. 11 was fitted to the nine species in the original database [13] for both response variables (PNB and TNB). The expected value and variance of the model parameters (${\beta }_{1}, \theta ,$ and $\sigma$) were computed from their marginal posterior probability distributions (Table 1; Supplementary Note 1) for the PNB and TNB. Subsequently, we used the estimated expected values and variances to determine the parameters of the probability distribution of ${\beta }_{1}, \theta ,$ and $\sigma$ via moment matching (Supplementary Note 2). Those parameters are reported in Table 3, facilitating their use as priors in future applications of the method presented in this study.

**Table 3:** Moments and hyperparameters for the model parameter for Partial N Balance (PNB) and Total N Balance (TNB)

Species	Parameter	Moments				Probability distribution	Hyper parameter	
		PNB		TNB				
		E(x)	Var(x)	E(x)	Var(x)		PNB	TNB
Blue lupin	${\beta }_{1}$	1.98	0.616	3.17	0.310	Gamma	(6.36, 3.21)	(32.41, 10.22)
	$\theta$	0.592	0.0063	0.569	0.0007	Beta	(22.10, 15.23)	(198.77, 150.56)
	$\sigma$	57.58	128.40	20.10	26.68	Gamma	(25.82, 0.45)	(15.14, 0.75)
chickpea	${\beta }_{1}$	0.70	0.014	1.72	0.032	Gamma	(35.00, 50.00)	(92.45, 53.75)
	$\theta$	0.644	0.0037	0.308	0.0007	Beta	(39.26, 21.70)	(93.47, 210.01)
	$\sigma$	19.63	5.76	25.59	12.56	Gamma	(66.90, 3.41)	(52.14, 2.04)
Common bean	${\beta }_{1}$	1.15	0.0912	1.23	0.083	Gamma	(14.50, 12.61)	(18.23, 14.82)
	$\theta$	0.574	0.0043	0.511	0.0022	Beta	(32.07, 23.80)	(57.53, 55.05)
	$\sigma$	24.46	14.29	22.98	12.78	Gamma	(41.87, 1.71)	(41.32, 1.80)
Cowpea	${\beta }_{1}$	1.53	0.084	2.81	0.328	Gamma	(27.87, 18.21)	(24.07, 8.57)
	$\theta$	0.542	0.0085	0.284	0.0048	Beta	(15.28, 12.92)	(11.74, 29.61)
	$\sigma$	23.36	7.92	60.76	51.66	Gamma	(68.90, 2.95)	(71.46, 1.17)
Faba bean	${\beta }_{1}$	1.61	0.048	2.76	0.110	Gamma	(54.00, 33.54)	(69.25, 25.09)
	$\theta$	0.609	0.0007	0.454	0.0013	Beta	(206.55, 132.61)	(86.11, 103.56)
	$\sigma$	33.06	7.20	51.36	17.13	Gamma	(151.80, 4.59)	(153.99, 3.00)
Field pea	${\beta }_{1}$	1.31	0.035	1.88	0.049	Gamma	(49.03, 37.43)	(72.13, 38.36)
	$\theta$	0.585	0.0008	0.449	0.0009	Beta	(176.94, 125.52)	(122.97, 150.91)
	$\sigma$	38.07	7.33	46.74	10.87	Gamma	(197.72, 5.19)	(200.98, 4.30)
Lentil	${\beta }_{1}$	0.94	0.021	1.31	0.042	Gamma	(42.07, 44.76)	(40.86, 31.19)
	$\theta$	0.637	0.0006	0.428	0.0025	Beta	(244.85, 139.53)	(41.48, 55.44)
	$\sigma$	18.55	2.74	26.62	5.48	Gamma	(125.58, 6.77)	(129.31, 4.86)
Peanut	${\beta }_{1}$	2.17	0.143	2.36	0.130	Gamma	(32.93, 15.17)	(42.84, 18.15)
	$\theta$	0.581	0.0008	0.515	0.0007	Beta	(176.22, 127.08)	(183.25, 172.57)
	$\sigma$	39.56	19.78	38.62	18.19	Gamma	(79.12, 2.00)	(81.99, 2.12)
White lupin	${\beta }_{1}$	2.41	0.217	3.17	0.310	Gamma	(26.76, 11.11)	(32.41, 10.22)
	$\theta$	0.859	0.0007	0.569	0.0007	Beta	(147.77, 24.25)	(198.77, 150.56)
	$\sigma$	23.63	37.53	20.09	26.68	Gamma	(18.89, 0.71)	(15.13, 0.75)

The moments were obtained from the posterior probability distribution and the hyperparameters were calculated using moment matching based on the previously obtained moments. For the gamma distributions, the first value between the parenthesis in the hyperparameter columns is the shape parameter ($\alpha$) while the second is the rate parameter ($\beta$). For the beta distributions those values are called shape ($\alpha$ and $\beta$) The hyperparameters might be used as priors in future applications of the model presented in this study

### Case study

In this section, we bring and describe a case study to showcase a potential use of the method presented in this article. This illustration is applied after estimating $\theta$, and quantifying its uncertainty, to formally determine whether the PNB underestimates the true N balance in legume species. The first step is computing the PNB and TNB using independent observations. Then, these observed PNB and TNB are used to fit the Bayesian model presented in Eqs.8–11. Once the model is fitted, the posterior probability distribution for ${\theta }_{PNB}$ and ${\theta }_{TNB}$ are selected. The Ndfa comparison is made simply by subtracting the posterior probability distribution for ${\theta }_{PNB}$ and ${\theta }_{TNB}$ parameters. Then the quantiles of the desired credible interval are computed. Finally, it is observed whether zero is included in the credible interval of the distribution of the difference: (i) if zero is included, we conclude ${\theta }_{PNB}$ and ${\theta }_{TNB}$ are not different, (ii) if zero is not included, ${\theta }_{PNB}$ and ${\theta }_{TNB}$ are different.

For simplicity, we implemented this case study only for two species, chickpea, and common bean, using data from a literature review [13]. Before fitting the model, we split the data into half by randomly sampling the collected studies (without replacement), which resulted in five chickpea and two common bean studies per subset. Then PNB and TNB were computed in each subset independently. The data was split to make comparisons between ${\theta }_{PNB}$ and ${\theta }_{TNB}$. Next, we fitted the Bayesian model (Eq. 8 to Eq. 11) to these subsets within each species. The posterior probability distribution for ${\theta }_{PNB}$ and ${\theta }_{TNB}$ were obtained and subtracted. The 95% credible interval was determined by computing the 0.025 and 0.975 quantiles of the probability distribution of the difference. Finally, it was observed whether zero was included in the credible interval.

### Computation and reproducibility

The Bayesian model presented in Eq. 8 to Eq. 11 was fitted using a Markov Chain Monte Carlo (MCMC) algorithm called Non-U-Turn Sampling (NUTS). A total of 4 chains were implemented with 20,000 iterations in total and 10,000 iterations as warm-up. The convergence of the chains was assessed visually through trace plots and analytically via the Gelman-Rubic diagnostic [22]. A seed was set for reproducibility. We performed the Bayesian analyses using Stan probabilistic programming language via rstan package [23]. The bootstrapping technique was implemented using the rsample package [24], while the delta method was carried out with the msm package [25]. All the statistical analyses were performed using the R software [26] in RStudio interface [27]. The code for the analyses is publicly available in https://github.com/FranciscoPalmero/Ndfa_uncertainty and https://figshare.com/s/60a9cf527ecb9de02166. The databases used in this article are available at https://figshare.com/s/60a9cf527ecb9de02166.

## Results

### Delta method, bootstrapping, and Bayesian inference performance

**Fig. 3:**
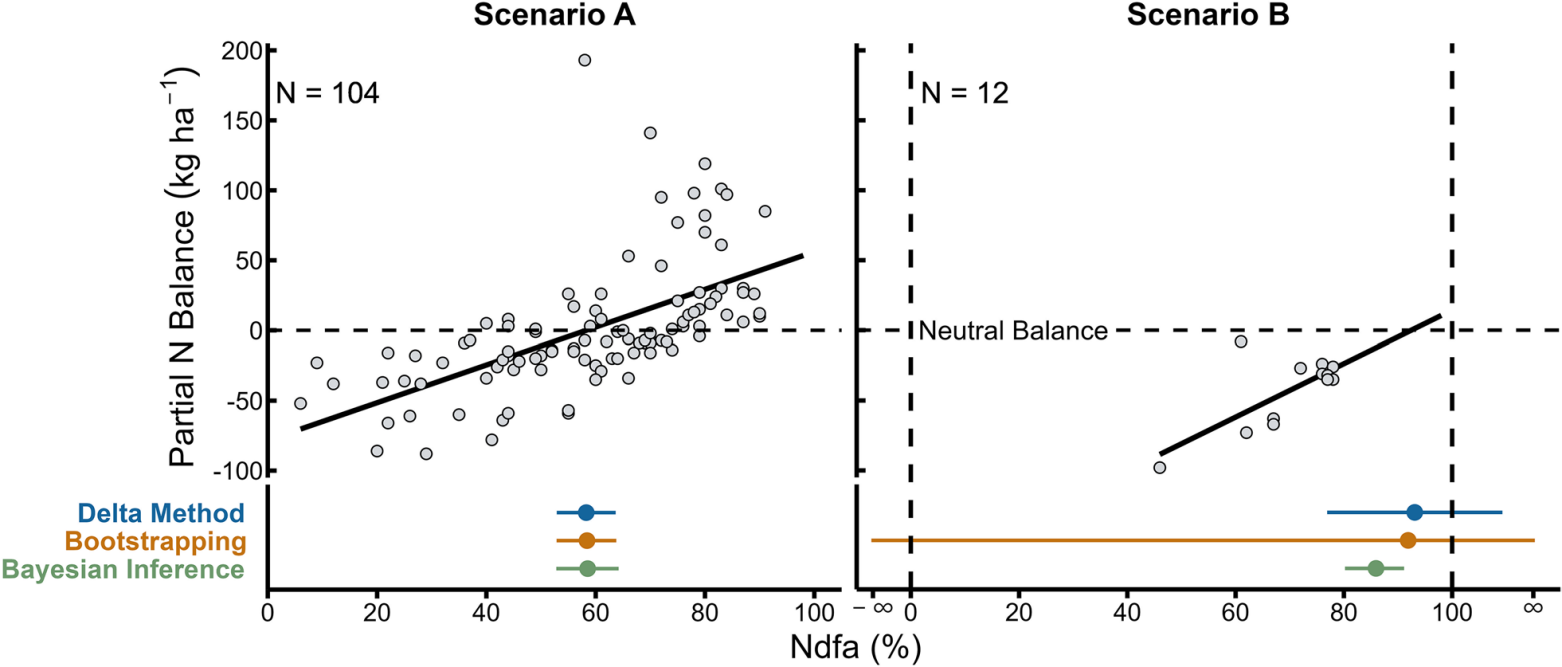
Point estimation and uncertainty quantification of $\theta$ implementing delta method, bootstrapping and Bayesian inference under two contrasting scenarios. In scenario A, the range of possible values for Ndfa is thoroughly explored (e.g., 6–91%), and the number of observations is relatively high ($n \ge 100$), and the three methods work similarly. In scenario B, observations of Ndfa are concentrated closer to its upper limit, the range of the Ndfa is poorly explored (e.g., 46–78%), and the number of observations is low (approximately 10), delta method and bootstrapping provide confidence intervals of $\theta$ that contain nonviable values in the real world. Field pea (*Pisum sativum* L.) (Scenario A) and white lupin (*Lupinus albus* L.) (Scenario B) data from [13] were implemented

We evaluated the delta method, bootstrapping, and Bayesian inference in two contrasting scenarios. These methods provided nearly equivalent numerical values when the range of possible values for Ndfa was thoroughly explored (e.g., 6–91%), and the number of observations was relatively high (e.g., $n\ge 100$) (Scenario A in Fig. 3). In the opposite case (Scenario B in Fig. 3) was when the Ndfa observations were closer to their upper limit, the range of the Ndfa was poorly explored (e.g., 46–78%), and the number of observations was low. Under these conditions, the delta method and bootstrapping provided uncertainty estimates, such as confidence intervals, that contained nonviable values in the real world, i.e. less than 0% or more than 100% (Scenario B in Fig. 3). Therefore, these results show that, in a data deficiency scenario, bootstrapping and delta method could yield values outside the expected biological range, 0–100%.

### Posterior probability distributions

**Fig. 4:**
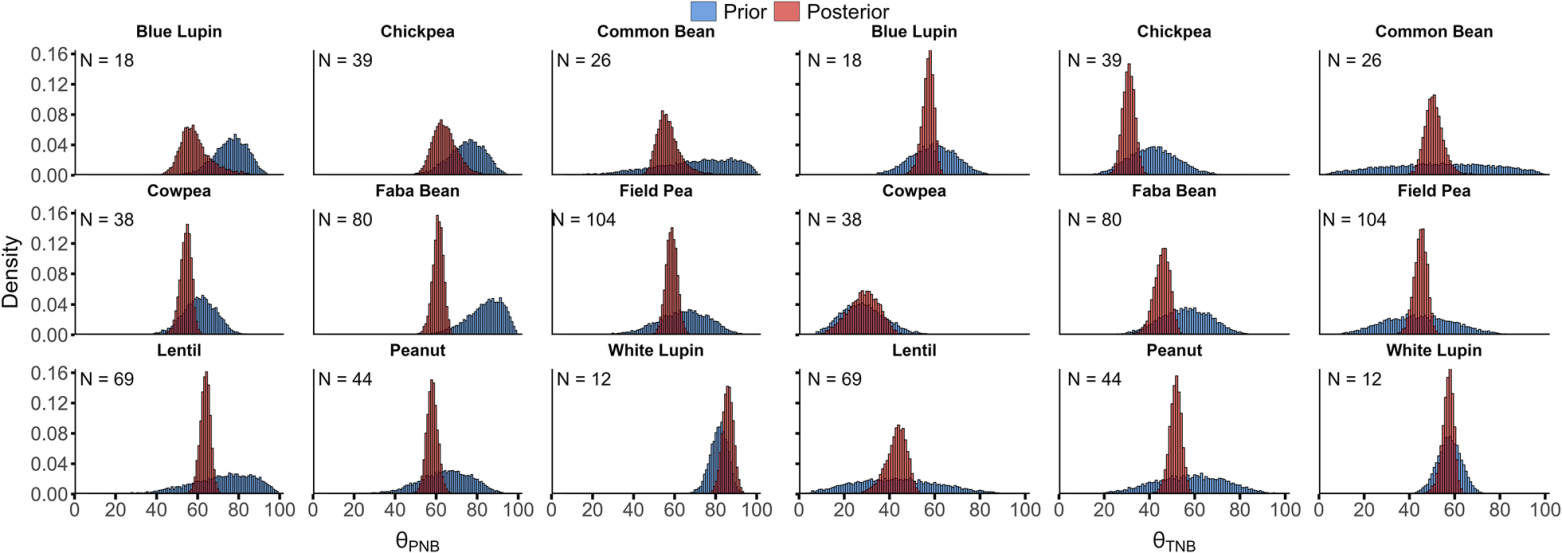
Histograms of samples from the prior (blue) and posterior (posterior) probability distributions for the $\theta$ parameter for Partial N Balance (${\theta }_{PNB}$) and Total N Balance (${\theta }_{TNB}$). The numbers inside the plot of each species indicate the number of observations. Priors are beta probability distributions based on the previously collected data from the literature, and posterior probability distributions were obtained as the marginal distributions of ${\theta }_{PNB}$ and ${\theta }_{TNB}$ via MCMC sampling

The expected value and variance of the model parameters for the nine species, computed from their marginal posterior probability distributions, are shown in Table 2. The $\theta$ parameter values fell within the biologically plausible range of [0, 100] (Fig. 4). This depicts how priors act as regulators in Bayesian models.

Initially, the hyperparameters for ${\beta }_{1}$ and $\sigma$ in the gamma distribution were the same across species. However, those values are now different, which is also the case for the parameters of the beta distributions for $\theta$ (see Table 2 and Table 3). This depicts how Bayesian framework can be used to combine prior knowledge (expressed as probability distributions) with the observed data to update our understanding about a given process in nature using concepts from probability theory. The influence of the observed data on the posterior probability distribution (updating process) depended on the number of observations for a given species. The greater the number of observations, the lower the influence of the prior on the posterior. This is depicted in Fig. 4 based on the proximity of the prior and posterior distribution peaks (the closer the peaks, the higher is the influence of the prior on the posterior).

We obtained the expected value and the variance of the model parameters from their marginal posterior probability distributions and utilized them to calculate the hyperparameters of their respective assumed distributions (priors). Therefore, the probability distributions shown in the last three columns of Table 3 can be used as prior distributions on future applications of the model presented in this study.

### Case study

**Fig. 5:**
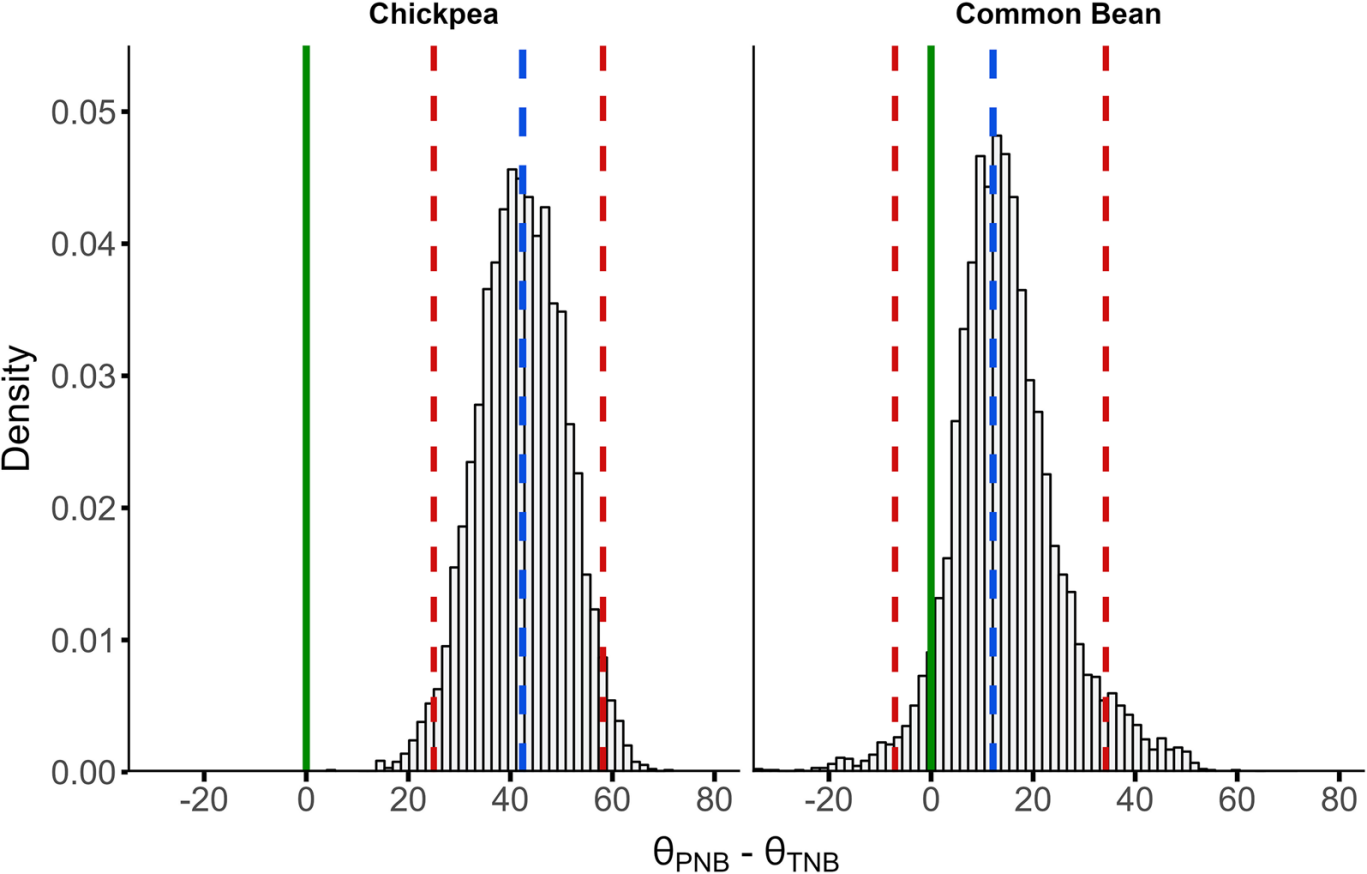
Posterior probability distribution of the difference between $\theta$ for Partial N Balance (${\theta }_{PNB}$) and Total N Balance (${\theta }_{TNB}$) in chickpea and common bean. The red dashed lines indicate the 0.025 and 0.975 quantiles, and the blue dashed lines indicate the median (0.5 quantile) of the posterior probability of the difference. The green solid lines indicate zero

A potential use of the proposed method was applied to chickpea and common bean to determine differences between ${\theta }_{PNB}$ and ${\theta }_{TNB}$. This case study is graphically represented in Fig. 5. The probability distribution for the difference between ${\theta }_{PNB}$ and ${\theta }_{TNB}$ are depicted through histograms. The lower and upper limits of the confidence interval were indicated with red-dashed lines. For chickpea, the absence of zero in the credible interval suggests a significantly lower Ndfa requirement for neutral N balance when belowground plant N is considered (Fig. 5). Specifically, chickpeas would require fixing between 22 and 58% less N, with a median of 41%, to achieve a neutral N balance if roots were considered. In contrast, for Common Bean, the N contribution from belowground plant structures was not substantial enough to show a significant difference in Ndfa requirement for neutrality (zero was included in the 95% credible interval; Fig. 5).

When interpreting this analysis, two main aspects should be noted: (i) The direction of the difference between ${\theta }_{PNB}$ and ${\theta }_{TNB}$. In this case, the difference was computed as ${\theta }_{PNB}- {\theta }_{TNB}$. This means that positive values indicate a lower Ndfa needed to achieve a neutral N balance when belowground plant N is considered. If the difference is computed as ${\theta }_{TNB}-{\theta }_{PNB}$, the conclusions about the significance of the difference are not affected (if zero is included or not), but the interpretation of the values is different. (ii) This method employs Bayesian inference, using credible intervals instead of confidence intervals common in classical analyses. The confidence intervals are understood as the proportion of experiments that would contain the true difference under a long series of replications of the experiment. While the credible interval is understood as the probability of the difference lying between the limits of the interval.

## Discussion

In this study, we developed a Bayesian framework to quantify the uncertainty on the Ndfa required to achieve neutral N balances in grain legume species. This approach was contrasted with the use of delta method and bootstrapping. The developed a framework allowed us to solve common issues in fitting N balance models, like obtaining unrealistic estimates and results, especially when using small datasets. This is the first study introducing new perspectives on using Bayesian inference for reliable estimations and uncertainty quantification of the Ndfa needed to achieve neutral PNB (${\theta }_{PNB}$) or TNB (${\theta }_{TNB}$) in grain legume species. We demonstrated the incorporation of previous knowledge into the model, as well as providing information for improving the inference in future studies. The hyperparameters of the posterior probability distribution of model parameters were presented, which can be implemented as prior in future investigations [28]. This article can also serve as a practical guide for agricultural scientists new to the Bayesian framework. Moreover, a case study demonstrated a potential application of the method, showcasing its usefulness in global estimations of N fixation contributions in grain legume species.

By applying the Bayesian framework, the probability distribution of $\theta$ was obtained for each of the addressed legume species. Previous studies analyzing the importance of grain legumes to N balance in agroecosystems reported point estimations for $\theta$ [7, 8, 13, 14]. The lack of uncertainty quantification does not enable researchers to make formal statistical inference. Therefore, the Bayesian framework proposed in this study opens new avenues for studies determining the role of grain legumes in nutrient balances in agroecosystems. Obtaining the probability distribution of $\theta$ provides us with complete information about the minimum Ndfa needed to achieve neutral PNB or TNB in grain legume species. Therefore, the Bayesian framework developed in this article lays the foundation not only to conduct formal comparisons or hypothesis testing involving $\theta$, but also to learn about its expected value, variance, and higher moments such as skewness and kurtosis under different agroecological, soil, and crop management conditions.

Beyond Bayesian inference, other statistical techniques were explored to quantify the uncertainty of $\theta$. One alternative was implementing the delta method to approximate the variance and confidence interval of $\theta$ [17]. Additionally, bootstrapping [10] was also utilized to obtain the empirical confidence intervals of $\theta$. Under limited data conditions, these two methods provided confidence intervals for $\theta$ that contain nonviable values in the real world. Usually, in non-Bayesian statistical inference, procedures are evaluated under asymptotic behavior, i.e. under large sample sizes. Since the delta method is justified under asymptotic conditions [29], it is expected that this method will not be consistent and efficient under small sample sizes, leading to an unreliable estimation of the uncertainty of $\theta$ under low data availability. Furthermore, although the delta method tends to underestimate the standard errors in comparison to bootstrapping [30], bootstrapped confidence intervals can still be erratic for small sample sizes [10].

Regularization techniques (Table 1) can be implemented to solve the issue of obtaining extremely wide or unrealistic confidence intervals on the estimation of a model parameter [18]. Although regularization techniques (e.g. ridge and Lasso regressions, and random effects in linear mixed models) might introduce bias on the estimation of the model parameters (e.g. ${\beta }_{1}$ and $\theta$), these techniques reduce the variance of those estimations, thereby narrowing the confidence intervals of the estimated parameters. However, the classical regularization approach does not provide guidance to select the penalization term [18, 31]. Iterative cross-validation can be implemented to select appropriate regulator parameters [18]. Nevertheless, this approach depends on out-of-sample data, which is an undesirable characteristic under limited data conditions. As well as, using cross-validation for determining the penalization term still yields point estimates for the model parameters, making it difficult or even impossible to quantify their uncertainty [18].

In the developed framework, we applied Bayesian inference for estimating and quantifying uncertainty while constraining (regularizing) the estimation of the model parameters via informative priors (regulator). Therefore, with respect to the classical regularization perspective (penalized likelihood), Bayesian models have the advantage of (i) providing formal guidance to define the hyperparameters for the priors (regulator), and (ii) utilizing formal probability theory for constraining the model parameters [31]. Priors can be selected so that the influence of the prior on the posterior is minimized (which are called weakly informative priors) or based on the prior knowledge about the parameters (informative priors) [28]. In this study, the use of informative priors, mainly under limited data conditions, enabled to: (i) combine independent datasets (collected from previous studies) into a simple modeling framework to obtain meaningful inference, and (ii) formally constrain the model parameters while estimating their uncertainty as it was previously depicted in ecology-related studies [20, 32].

In this study, we showed that the developed Bayesian framework excelled under limited data conditions as it was shown in other field of studies such as epidemiology and medicine [33, 34]. However, the power of Bayesian inference under low number of observations was paid by adding stronger assumptions into the model, which were the use of informative priors. The informative priors must be correctly justified by collecting information from sources such as literature, previous experiments, expert knowledge, natural or biological conditions, among others to make the inference reliable [11]. Furthermore, the developed Bayesian framework was worthwhile when the collected data were close to biological limits and the model parameter estimations had to be regularized to obtain meaningful inference [18]. Under large sample sizes, the influence of the priors on the posterior is reduced [35], the delta method improves its consistency and efficiency because of their asymptotic behavior [36, 37], and the bootstrapped confidence intervals are more consistent [10]. Therefore, with a high number of observations, the developed Bayesian framework, bootstrapping, and delta method provided similar uncertainty quantification of $\theta$.

Bayesian inference is an available statistical tool that seems to be underutilized in agriculture studies addressing the N contribution by legumes and the estimation of $\theta$. The framework presented in this article can also be applied to gain knowledge about the maximum N output that a grain legume species can achieve to contribute with N to the system. Furthermore, this Bayesian framework can also be implemented to obtain the distribution of the minimum N uptake needed before a legume crop is able to start fixing N [13, 21, 38]. However, the use of Bayesian inference in agriculture transcends these potential applications in the field of N fixation in grain legume species. Bayesian inference is a pertinent tool in agricultural sciences, where uncertainty is present everywhere, and previous and expert knowledge hold significant importance. Therefore, we hope this article also serves as an initial guide for Bayesian non-practitioners in the field of agriculture to apply Bayesian inference (regarding that other approaches can also be valid).

The databases and the code utilized in this study have been made publicly available. Science utilizes data (evidence) to answer accurate questions (hypotheses) by inductive reasoning developing new knowledge or theories that are then used as a benchmark to formulate further questions that are proved or disproved in future studies [39]. Therefore, science is a continuous built from a community sharing its knowledge. By making the utilized databases and codes publicly available, our objective was to contribute to the development of a more robust, transparent, and reliable approach to advancing scientific knowledge [40]. The available datasets can be implemented to test new hypotheses related to N balance in legume species, such as whether the contribution by roots is substantial in a given species (differences between ${\theta }_{PNB}$ and ${\theta }_{TNB}$), identify potential changes in the PNB or TNB per unit of Ndfa among species or within species (differences in ${\beta }_{1}$), or analyzing the uncertainty around relative N outputs (i.e. NHI) between and within species. Building such datasets demands a significant investment of time and effort. Consequently, we seek to engage additional collaborators in the ongoing process of database updating. This entails the incorporation of new studies, additional N inputs and outputs (e.g., N_2_O and/or NH_2_ emissions, NO_2_ leaching), and the addition of metadata, including crop rotation, weather conditions, topography, and tillage practices, among other variables.

Simplified N balances of the legume crops were implemented in this study to estimate the uncertainty of $\theta$. Although this a common a practice to evaluate the N benefit of grain legume species to cropping systems [7, 8, 13, 14, 38, 41–43], this approach does not consider other N inputs and losses [5, 6], leading to oversimplified N balance estimations. Therefore, upcoming research should study $\theta$ considering other N inputs and outputs beyond the fixed N and the N exported in seeds, respectively, to better understand the role of grain legume species in the N balance of the agroecosystems. Furthermore, there exist different techniques to measure the N contribution of belowground components [15]. The variety of techniques along with the difficulty in recovering nodules, and thin and fragile roots generate uncertainty on the N contribution of belowground components and consequently on the root factor [13]. Hence, subsequent studies should account for this variability on belowground N contribution when computing the uncertainty of ${\theta }_{TNB}$. Additionally, a higher quantity and quality of data to estimate the proportion of Ndfa derived from roots are needed to improve the accuracy of the prior information to be included in the presented Bayesian framework.

## Conclusion

This study explored the use of the delta method, bootstrapping, and Bayesian inference to quantify the uncertainty of the Ndfa that grain legume species need to attain neutral PNB or TNB ($\theta$). For Bayesian models, regularization is a natural consequence of using informative priors. This article depicted the usefulness of Bayesian approach to obtain meaningful inference and formally constrain the model parameters via the combination of independent data sets into the same modeling framework. Since there exists knowledge about the Ndfa needed to achieve neutral PNB or TNB in grain legume species, we expect that the use of informative priors takes more relevance when estimating this quantity and its uncertainty. Future studies should provide information of the slope (${\beta }_{1}$) and the Ndfa needed to achieve neutral PNB or TNB ($\theta$) to develop informative priors, being crucial to fully embrace the potential of regularization. The developed Bayesian inference framework can be transferred to estimate balances for other nutrients and/or field crops to gain more knowledge on global crop nutrient balances.

### Supplementary Information

Additional file 1

## Data Availability

The datasets and the R code utilized in the current study are freely available at https://figshare.com/s/60a9cf527ecb9de02166. The R code is also available at https://github.com/FranciscoPalmero/Ndfa_uncertainty/blob/main/Ndfa_uncestim_011624.Rmd.

## References

[1] Jensen ES, Carlsson G, Hauggaard-Nielsen H. Intercropping of grain legumes and cereals improves the use of soil N resources and reduces the requirement for synthetic fertilizer N: a global-scale analysis. Agron Sustain Dev. 2020;40:5. 10.1007/s13593-020-0607-x

[2] Jensen ES, Peoples MB, Boddey RM, Gresshoff PM, Hauggaard-Nielsen H, J.R. Alves B, et al. Legumes for mitigation of climate change and the provision of feedstock for biofuels and biorefineries a review. Agron Sustain Dev. 2012;32:329–64. 10.1007/s13593-011-0056-7

[3] Haynes RJ, Martin RJ, Goh KM. Nitrogen fixation, accumulation of soil nitrogen and nitrogen balance for some field-grown legume crops. Field Crop Res. 1993;35:85–92. 10.1016/0378-4290(93)90141-9

[4] Corre-Hellou G, Crozat Y. N2 fixation and N supply in organic pea (Pisum sativum L.) cropping systems as affected by weeds and peaweevil (Sitona lineatus L.). European J Agronomy. 2005;22:449–58. 10.1016/j.eja.2004.05.005

[5] Ross SM, Izaurralde RC, Janzen HH, Robertson JA, McGill WB. The nitrogen balance of three long-term agroecosystems on a boreal soil in western Canada. Agr Ecosyst Environ. 2008;127:241–50. 10.1016/j.agee.2008.04.007

[6] Eickhout B, Bouwman AF, van Zeijts H. The role of nitrogen in world food production and environmental sustainability. Agr Ecosyst Environ. 2006;116:4–14. 10.1016/j.agee.2006.03.009

[7] Gollner G, Starz W, Friedel JK. Crop performance, biological N fixation and pre-crop effect of pea ideotypes in an organic farming system. Nutr Cycl Agroecosyst. 2019;115:391–405. 10.1007/s10705-019-10021-4

[8] Kehoe E, Rubio G, Salvagiotti F. Contribution of different sources and origins of nitrogen in above- and below-ground structures to the partial nitrogen balance in soybean. Plant Soil. 2022;477:405–22. 10.1007/s11104-022-05418-0

[9] Ver Hoef JM. Who invented the delta method? Am Stat. 2012;66:124–7. 10.1080/00031305.2012.687494

[10] Efron B, Tibshirani R. Bootstrap methods for standard errors, confidence intervals, and other measures of statistical accuracy. Stat Sci. 1986;1:54–75.

[11] McElreath R. Statistical Rethinking: a bayesian course with examples in R and STAN. 2nd ed. Boca Raton: CRC Press; 2020.

[12] Fienberg SE. When did Bayesian inference become “Bayesian”? Bayesian Anal. 2006. 10.1214/06-BA101. 10.1214/06-BA101

[13] Palmero F, Fernandez JA, Garcia FO, Haro RJ, Prasad PVV, Salvagiotti F, et al. A quantitative review into the contributions of biological nitrogen fixation to agricultural systems by grain legumes. Eur J Agron. 2022;136: 126514. 10.1016/j.eja.2022.126514

[14] Walley FL, Clayton GW, Miller PR, Carr PM, Lafond GP. Nitrogen economy of pulse crop production in the northern great plains. Agron J. 2007;99:1710–8. 10.2134/agronj2006.0314s

[15] Fustec J, Lesuffleur F, Mahieu S, Cliquet J-B. Nitrogen rhizodeposition of legumes. A review Agron Sustain Dev. 2010;30:57–66. 10.1051/agro/2009003

[16] Carranca C, Torres MO, Madeira M. Underestimated role of legume roots for soil N fertility. Agron Sustain Dev. 2015;35:1095–102. 10.1007/s13593-015-0297-y

[17] Powell LA. Approximating variance of demographic parameters using the delta method: a reference for avian biologists. The Condor. 2007;109:949–54. 10.1093/condor/109.4.949

[18] Hooten MB, Hobbs NT. A guide to Bayesian model selection for ecologists. Ecol Monogr. 2015;85:3–28. 10.1890/14-0661.1

[19] Gelman A, Carlin JB, Stern HS, Dunson DB, Vehtari A, Rubin DB. Bayesian data analysis. 3rd ed. Boca Raton: CRC Press; 2013.

[20] Hobbs NT, Hooten MB. Bayesian models: a statistical primer for ecologists. Princeton, New Jersey: Princeton University Press; 2015.

[21] Anglade J, Billen G, Garnier J. Relationships for estimating N _2_ fixation in legumes: incidence for N balance of legume-based cropping systems in Europe. Ecosphere. 2015;6:1–24. 10.1890/ES14-00353.1

[22] Gelman A, Rubin DB. Inference from Iterative simulation using multiple sequences. Stat Sci. 1992;7:457–72. 10.1214/ss/1177011136

[23] Stan DT. RStan: the R interface to Stan. https://mc-stan.org/ 2022.

[24] Frick H, Chow F, Kuhn M, Mahoney M, Silge J, Wickham H. rsample: General Resampling Infrastructure. 2022.

[25] Jackson C. Multi-state models for panel data: the MSM package for R. J Stat Softw. 2011;38:1–28. 10.18637/jss.v038.i08

[26] R Core Team. R: a language and environment for statistical computing. R Foundation for Statistical Computing: Vienna, Austria; 2020.

[27] Posit team. RStudio: integrated development environment for R. Boston, MA: Posit Software; 2023.

[28] Gelman A. Prior distributions for variance parameters in hierarchical models (comment on article by Browne and Draper). Bayesian Anal. 2006;1:515–34. 10.1214/06-BA117A

[29] Beutner E. Delta method, asymptotic distribution. WIREs Comput Stat. 2024;16: e1634. 10.1002/wics.1634

[30] Efron B. Six questions raised by the bootstrap (Technical Report No. 139). Stanford, CA: Stanford University, Division of Biostatistics; 1990.

[31] Hefley TJ, Hooten MB. On the existence of maximum likelihood estimates for presence-only data. Methods Ecol Evol. 2015;6:648–55. 10.1111/2041-210X.12340

[32] Hefley TJ, Hooten MB, Drake JM, Russell RE, Walsh DP. When can the cause of a population decline be determined? Ecol Lett. 2016;19:1353–62. 27678091 10.1111/ele.12671

[33] Cook JD, Williams DM, Walsh DP, Hefley TJ. Bayesian forecasting of disease spread with little or no local data. Sci Rep. 2023;13:8137. 37208385 10.1038/s41598-023-35177-6 PMC10199067

[34] Dexter F, Ledolter J. Bayesian prediction bounds and comparisons of operating room times even for procedures with few or no historic data. Anesthesiology. 2005;103:1259–1167. 16306741 10.1097/00000542-200512000-00023

[35] van de Schoot R, Depaoli S, King R, Kramer B, Märtens K, Tadesse MG, et al. Bayesian statistics and modelling. Nat Rev Methods Primers. 2021;1:1. 10.1038/s43586-020-00001-2

[36] Weba M, Dörmann N. Application of the delta method to functions of the sample mean when observations are dependent. Stat Papers. 2017;58:957–86. 10.1007/s00362-015-0734-7

[37] Zepeda-Tello R, Schomaker M, Maringe C, Smith MJ, Belot A, Rachet B, et al. The delta-method and influence function in medical statistics: a reproducible tutorial. arXivorg. 2022. 10.48550/arXiv.2206.15310. 10.48550/arXiv.2206.15310

[38] Salvagiotti F, Cassman KG, Specht JE, Walters DT, Weiss A, Dobermann A. Nitrogen uptake, fixation and response to fertilizer N in soybeans: a review. Field Crop Res. 2008;108:1–13. 10.1016/j.fcr.2008.03.001

[39] Howson C, Urbach P. Scientific reasoning: the bayesian approach. 3rd ed. Chicago: Open Court; 2005.

[40] Nosek BA, Alter G, Banks GC, Borsboom D, Bowman SD, Breckler SJ, et al. Promoting an open research culture. Science. 2015;348:1422–5. 26113702 10.1126/science.aab2374 PMC4550299

[41] Peoples MB, Herridge DF, Ladha JK. Biological nitrogen fixation: an efficient source of nitrogen for sustainable agricultural production? Plant Soil. 1995;174:3–28. 10.1007/BF00032239

[42] López-Bellido L, López-Bellido RJ, Redondo R, Benítez J. Faba bean nitrogen fixation in a wheat-based rotation under rainfed Mediterranean conditions: effect of tillage system. Field Crop Res. 2006;98:253–60. 10.1016/j.fcr.2006.03.001

[43] Ciampitti IA, Salvagiotti F. New Insights into soybean biological nitrogen fixation. Agron J. 2018;110:1185–96. 10.2134/agronj2017.06.0348

