# Supplementary material for: A Bayesian approach for estimating the uncertainty on the contribution of nitrogen fixation and calculation of nutrient balances in grain legumes
Source: Plant Methods. 2024 Sep 2;20:134. doi: 10.1186/s13007-024-01261-9 (PMC11367983; doi:10.1186/s13007-024-01261-9)
Supplement: Supplementary file 1 — Additional file 1 [file 13007_2024_1261_MOESM1_ESM.docx]

**Supplementary Note 1**

Marginal posterior probability distributions for each of the model parameters. The bracket notation “[.]” indicates a probability density distribution. The marginal posterior probability distributions are obtained by integrating over all other variables in the joint posterior ($\left[ y|\beta_{1},\theta,\sigma\right]\left[ \beta_{1} \right]\left[ \theta\right]\left[ \sigma\right]$).

$\left[ \beta_{1}|. \right]\propto\int_{0}^{\infty} \int_{0}^{1} \left[ y|\beta_{1}, \theta, \sigma\right]\left[ \beta_{1} \right]\left[ \theta\right]\left[ \sigma\right] d\theta d\sigma= \left[ y|\beta_{1},\theta,\sigma\right]\left[ \beta_{1} \right]$

$\left[ \theta|. \right]\propto\int_{0}^{\infty} \int_{0}^{\infty} \left[ y|\beta_{1}, \theta, \sigma\right]\left[ \beta_{1} \right]\left[ \theta\right]\left[ \sigma\right] d\beta_{1}d\sigma= \left[ y|\beta_{1},\theta,\sigma\right]\left[ \theta\right]$

$\left[ \sigma|. \right]\propto\int_{0}^{\infty} \int_{0}^{1} \left[ y|\beta_{1}, \theta, \sigma\right]\left[ \beta_{1} \right]\left[ \theta\right]\left[ \sigma\right] d\theta d\beta_{1}= \left[ y|\beta_{1},\theta,\sigma\right]\left[ \sigma\right]$

**Supplementary Note 2**

Probability Distributions: Moments and Moment Matching

Probability distributions can be specified and summarized using what is known as moments of the distribution (**Table 1**; [1]). The first moment and the second central moment of a random variable are well known since they represent its expected value (mean) and variance, respectively. Therefore, the first moment summarizes the mean of the distribution of a random variable, while the second central moment provides an intuitive measure of the spread around the mean. These probability distributions are shaped by specific parameters that, usually, are functions of the first moment and the second central moment. Thus, it is usually not possible to directly use the mean and the variance to fully describe the distribution except in special cases. This presents a challenge when we seek to describe the knowledge of parameters such as $\theta$, as we often think directly in terms of the mean and variance (or standard deviation) and not in terms of functions of these moments.

This challenge can be addressed through moment matching [1]. The parameters of the distribution are functions of the mean and variance. For instance, the gamma distribution has two parameters called $\alpha$ and $\beta$ (1.6 and 0.8 in Eq.9, respectively). These parameters can be written as $\alpha= \frac{\mu^{2}}{\sigma^{2}}$ and $\beta=\frac{\mu}{\sigma^{2}}$, where $\mu$ and $\sigma^{2}$ are the mean and the variance of the distribution, respectively. Therefore, it is possible to express these moments as a function of the parameters as $\mu= \frac{\alpha}{\beta}$ and $\sigma^{2}= \frac{\alpha}{\beta^{2}}$. Then, once we know $\mu$ and $\sigma^{2}$, we can simultaneously solve for $\alpha$ and $\beta$ in $\mu= \frac{\alpha}{\beta}$ and $\sigma^{2}= \frac{\alpha}{\beta^{2}}$ to compute the values of $\alpha$ and $\beta$ that give the known $\mu$ and $\sigma^{2}$. This example illustrates that, via moment matching, it is possible to come up with the parameters of the distribution by initially knowing the mean and the variance of a random variable.

**
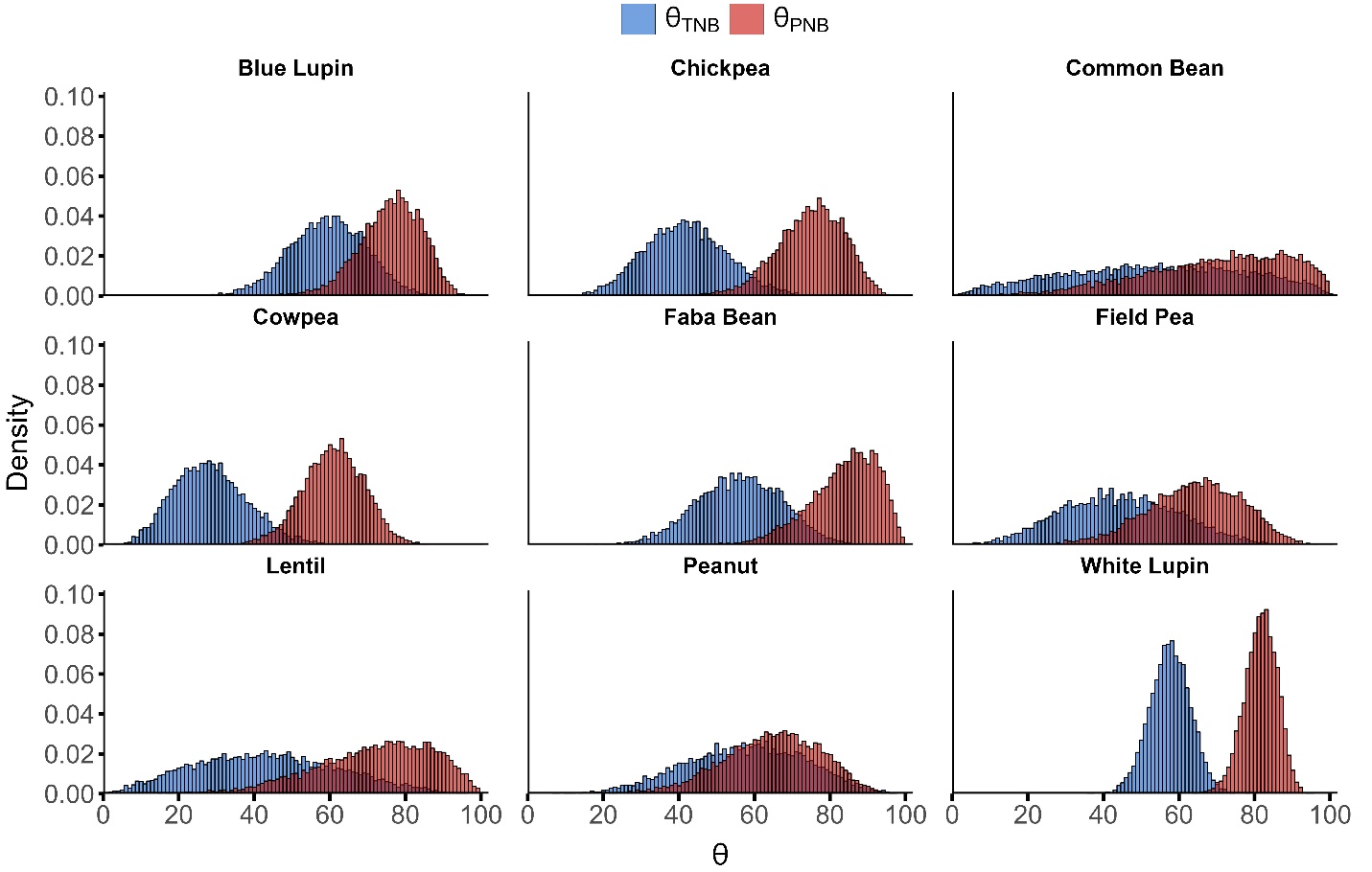
Supplementary Figure 1**. Prior probability distributions for $\theta_{PNB}$ (red) and $\theta_{TNB}$ (blue) for each legume species. In both cases ($\theta_{PNB}$ and $\theta_{TNB}$) the prior probability distributions were beta distributions which were scaled to get values in the interval [0,100].

**References**

1. Hobbs NT, Hooten MB. Bayesian models: a statistical primer for ecologists. Princeton, New Jersey: Princeton University Press; 2015.
